# Changes in Policy Supports for Healthy Food Retailers, Farmers Markets, and Breastfeeding Among US Municipalities, 2014–2021: National Survey of Community-Based Policy and Environmental Supports for Healthy Eating and Active Living (CBS-HEAL)

**DOI:** 10.5888/pcd20.230018

**Published:** 2023-08-17

**Authors:** Stephen J. Onufrak, Latetia V. Moore, Samantha L. Pierce, Carol A. MacGowan, Deborah A. Galuska

**Affiliations:** 1 Division of Diabetes Translation, National Center for Chronic Disease Prevention and Health Promotion, Centers for Disease Control and Prevention, Atlanta, Georgia; 2 Division of Nutrition, Physical Activity, and Obesity, National Center for Chronic Disease Prevention and Health Promotion, Centers for Disease Control and Prevention, Atlanta, Georgia

## Abstract

**Introduction:**

Policies and practices at the local level can help reduce chronic disease risk by providing environments that facilitate healthy decision-making about diet.

**Methods:**

We used data from the 2014 and 2021 National Survey of Community-Based Policy and Environmental Supports for Healthy Eating and Active Living to examine prevalence among US municipalities of policies to support access to healthier food in supermarkets, convenience stores, and farmers markets, as well as policies to support breastfeeding among government employees. Chi-square tests were conducted to compare prevalence estimates from 2021 to 2014 overall and according to municipal characteristics.

**Results:**

In 2021, 29% of municipalities had at least 1 policy to encourage full-service grocery stores to open stores, which was not significantly different from 31% in 2014. Prevalence of having at least 1 policy to help corner stores sell healthier foods declined significantly from 13% in 2014 to 9% in 2021. Prevalence of policies providing all local government employees who were breastfeeding breaktime and space to pump breast milk increased significantly from 25% in 2014 to 52% in 2021. The percentage of municipalities that provided 8 or more weeks of paid maternity leave for employees increased significantly from 16% in 2014 to 19% in 2021.

**Conclusion:**

Prevalence of supports for supermarkets, convenience stores, and farmers markets generally did not increase among US municipalities from 2014 to 2021, while some supports for breastfeeding among municipal employees increased during this time. Opportunities exist to improve municipal-level policies that support healthy eating and breastfeeding among community residents and employees.

**Summary**

**What is already known on this topic?**

Local governments can use policies and practices to facilitate healthy nutrition and breastfeeding to help prevent chronic disease.

**What is added by this report?**

Between 2014 and 2021, the percentage of governments reporting policies to support healthy food retail in supermarkets, corner stores, and farmers markets did not substantially increase; however, some policies to support breastfeeding among government employees increased significantly.

**What are the implications for public health practice?**

Opportunities exist to improve municipal-level policies that support healthy eating and breastfeeding among community residents and employees.

## Introduction

The environments in which people live, learn, work, and play influence their ability to consume nutritious foods, access safe places for physical activity, and engage in other health-promoting behaviors, such as breastfeeding (1). These behaviors can reduce risk for chronic health conditions, including obesity, high blood pressure, type 2 diabetes, and certain cancers (2,3). Furthermore, breastfeeding confers short- and long-term health benefits for both mothers and infants (4). Policies and practices at the local, state, and federal levels can help improve nutritional risk factors for health by providing environments that facilitate healthy decision making (1). In 2009, the Institute of Medicine (IOM) and Centers for Disease Control and Prevention (CDC) released recommended strategies for communities and municipalities to prevent obesity and related chronic diseases through facilitating healthy eating, breastfeeding, and physical activity among residents (5,6). Some of these strategies include policies and practices aimed at increasing community access to healthy foods for supermarkets, convenience stores, and farmers markets. For example, local governments may encourage supermarkets to open in underserved areas, encourage existing corner or convenience stores to stock healthier foods, or facilitate access to farmers markets by streamlining operational processes or providing technical assistance, loans, or grants (5,6). Other recommended strategies promote and support optimal breastfeeding practices in the community and in government worksites (5,6). These strategies include policies to provide paid maternity leave to municipal employees and to permit breaktime and private spaces for breastfeeding employees to pump breast milk while at work (5,6).

Although many studies have examined and evaluated individual policies that aimed to improve access to healthy foods and support breastfeeding, less information exists on how commonly these policies and practices are found across the US. To ascertain the prevalence of policies and practices that support healthy eating, physical activity, and breastfeeding among US municipalities, CDC conducted a survey of municipal governments in 2014, the National Survey of Community-Based Policy and Environmental Supports for Healthy Eating and Active Living (CBS-HEAL). Using a nationally representative sample of US municipalities with a population of 1,000 or more, the CBS-HEAL study examined municipal policies and practices to support healthy eating and active living, including many recommended by IOM and CDC (5,6). Using data from CBS-HEAL, Lange et al found that two-thirds (67%) of US municipalities reported that they provided support for local farmers markets, while fewer provided support for supermarkets (34%) or convenience or corner stores (14%) (7). Of note, these healthy food retail policies were more common in larger municipalities (≥50,000 people) (7). These national data were important to set benchmarks, and they substantiated the notion that some local governments have taken action. They also suggested opportunities for municipalities to better support healthy decision making among community residents and employees.

In 2021, CDC conducted a second administration of CBS-HEAL using a similar sample design and survey items to the 2014 CBS-HEAL survey to enable comparisons and monitoring of progress in communities over time. Our analysis aimed to 1) document the national prevalence of municipal-level efforts to support healthy food retail (supermarkets, corner stores, and farmers markets) in communities and breastfeeding for government employees in 2021, overall and by municipality characteristics; and 2) compare the prevalence estimates from 2021 to the estimates from 2014.

## Methods

### Study design and population

The 2014 and 2021 CBS-HEAL surveys are nationally representative surveys of US municipalities with populations of 1,000 or more; similar sampling methods were used for both surveys. The sampled municipalities for 2014 and 2021 were drawn from the 2007 and 2017 US Census of Governments respectively, which were the most recent available at the time of each survey. The surveys used explicitly stratified sampling by US Census region (Northeast, Midwest, South, and West) and urban/rural status, which was defined on the basis of proportion of a Census place’s population that resides within a Census-designated urban area. Further implicit stratification, performed by sorting by population size, was also used in each stratum to ensure that small, medium, and large municipalities from each stratum were included in the sample. The survey was sent to the city or town manager, city planner, city administrator, or someone in a similar role in each municipality who could complete the survey through an electronic module or, if requested, paper- or interviewer-administered telephone survey. In 2014, 4,484 municipalities were sampled and 2,029 completed the survey, and in 2021, 4,417 municipalities were sampled and 1,982 completed the survey, both of which correspond to a response rate of 45%. Most (>80%) completed the survey via web. More information about the survey can be found at www.cdc.gov/nccdphp/dnpao/division-information/data-stats/cbs-heal/index.html. For this study, municipalities with missing data on any of the healthy food retail supports examined (n = 90; 35 from 2014 and 55 from 2021) or missing data on breastfeeding supports (n = 57; 15 from 2014 and 42 from 2021) were excluded from analyses of those variables.

### Variables

We examined policies and practices to support access to healthier foods in supermarkets, convenience stores, and farmers markets as well as policies to support government employee breastfeeding (Box). These policies were included on both the 2014 and 2021 surveys using very similar questions. In the context of this study, “policy supports” may encompass a range of policies, regulations, guidelines, programs, or practices to capture the diverse approaches municipal governments may take to support healthy nutrition in the framework of their local government.

**Box. Municipal Policies and Practices that Support Access to Healthy Foods in Supermarkets, Convenience Stores, and Farmers Markets and Policies to Support Breastfeeding Among Municipal Employees, Community-Based Policy and Environmental Supports for Healthy Eating and Active Living Survey, 2014 and 2021**

**Policies and practices to encourage supermarkets or full-service grocery stores to open stores**

• Tax incentives

• Grant or loan programs

• Programs to link store openings to broader revitalization projects

• [If Yes to Any Above] Do any of these policies or programs used by your community to encourage supermarkets and other full-service grocery stores to open stores explicitly prioritize low-income or under-resourced areas?

**Policies and practices to help convenience or corner stores sell healthier foods**

• Grant or low-interest loan programs to purchase equipment for storage or sales of healthful food

• Technical assistance or training programs to increase the ability to sell healthier foods

• Programs to link stores to broader neighborhood revitalization projects

• [If Yes] Do any of these policies or programs used to help convenience or corner stores sell healthier foods explicitly prioritize low-income or under-resourced areas?

**Policies and practices related to farmers’ markets, farm stands, or green/produce carts**

• Allow vendors to sell fresh produce on city-owned property

• Streamline processes for obtaining health or food safety permits and licenses

• Extend waivers of required business permits or retail licensing fees or taxes

• Provide funds or in-kind services for personnel, signage, or advertising

• Provide funding for Electronic Benefits Transfer (EBT) machines or provide technical assistance on how to obtain or use EBT machines

Municipal characteristics were derived from the 2013 and 2020 American Community Survey 5-year estimates (8). Characteristics included population size (1,000–2,499, 2,500–49,999, or ≥50,000), rural/urban status (based on whether ≥50% of population for a municipality resides in an urbanized area), US Census region (Northeast, Midwest, South, or West) (9), median educational attainment (≥some college or ≤high school graduate), percentage of the population living below the federal poverty level (<20% or ≥20%) to reflect persistent poverty as defined by the US Department of Agriculture (10), and racial/ethnic composition of the municipality (>50% non-Hispanic White or ≤50% non-Hispanic White).

### Analyses

The prevalence and 95% CI of each policy support for supermarkets, convenience stores, farmers markets, and employee breastfeeding was estimated for 2014 and 2021 using SAS survey procedures (SAS Institute) to account for design variables, nonresponse, and sample weights. We also assessed the prevalence of having at least 1 of any of the policy supports for each of the 3 domains: supermarkets, convenience stores, and farmers markets. Chi-square tests were used to compare prevalence between survey years with *P* < .05 set as significance. Among municipalities that reported at least 1 policy support for supermarkets or convenience stores, we also assessed the percentage of municipalities that indicated that the policy prioritized low-income or under-resourced areas and compared 2014 and 2021 prevalence using χ^2^ tests.

Finally, we assessed prevalence of having at least 1 policy support for each of the 3 retail domains (supermarkets, convenience stores, and farmers markets) and breastfeeding supports according to municipal characteristics. Chi-square tests were performed to assess whether prevalence of each policy support differed between 2014 and 2021 for each level of municipal characteristic (eg, comparing the prevalence of any paid maternity leave between 2014 and 2021 among municipalities in the South).

For this study, municipalities who responded “don’t know” for a specific policy support were classified as having responded “no.” The median percentage of “don’t know” responses across all policies examined was 8.4% (range, 6.0% [“Technical assistance or training programs to increase the ability to sell healthier foods” in convenience or corner stores] to 21.6% [“Does your local government have a policy that allows ALL (salaried and hourly) local government employees breaktime and space to pump breast milk?”]). To investigate the potential impact of differing patterns of “don’t know” responses between survey years on results, we also performed sensitivity analyses where “don’t know” responses were excluded from calculations of overall prevalences and related statistical tests.

## Results

No significant differences were found between the 2014 and 2021 CBS-HEAL samples according to population size, rural/urban status, or Census region (Table 1). In both years, approximately one-third of municipalities had a population of 1,000 to 2,499; 58% to 59% had a population of 2,500 to 49,999; 7% had a population of 50,000 or more; and approximately one-quarter were rural. Regarding region, 14% to 15% of municipalities were in the Northeast, 35% were in the Midwest, 36% were in the South, and 15% were in the West. The 2014 and 2021 samples differed significantly in terms of education, poverty, and race and ethnicity. Compared with the 2014 sample, the 2021 sample had a higher percentage of municipalities whose residents had some college education (67.7% vs 55.4%, *P* < .01), a smaller percentage of municipalities with poverty prevalence of 20% or more (21.5% vs 30.4%, *P* < .01), and a greater percentage of municipalities that were 50% or less non-Hispanic White (16.3% vs 13.4%, *P* = .01).

**Table 1:** Characteristics of Participating Municipalities, National Survey of Community-Based Policy and Environmental Supports for Healthy Eating and Active Living (CBS-HEAL), 2014 and 2021^a^

Municipality characteristic (n)	Survey year		*P* value^b^
	2014 (n = 1,994) % (95% CI)	2021 (n = 1,927) % (95% CI)	
**Population size**			
1,000–2,499 (n = 1,367)	34.9 (33.1–36.8)	33.8 (31.8–35.8)	.73
2,500–49,999 (n = 2,259)	58.2 (56.8–61.1)	59.0 (56.8–61.1)	
≥50,000 (n = 295)	6.8 (5.8–7.9)	7.2 (6.1–8.3)	
**Rural/urban status**			
Urban (n = 2,875)	74.7 (73.2–76.1)	75.6 (74.0–77.2)	.50
Rural (n = 1,038)	25.3 (23.8–26.8)	24.4 (22.8–26.0)	
**Census region**			
Northeast (n = 523)	14.6 (13.3–15.9)	14.0 (12.8–15.1)	.95
Midwest (n = 1,383)	35.0 (33.6–36.5)	35.4 (33.8–37.0)	
South (n = 1,239)	35.9 (34.4–37.4)	35.9 (34.2–37.6)	
West (n = 776)	14.5 (13.4–15.6)	14.8 (13.8–15.7)	
**Median educational attainment**			
Some college or more (n = 2,458)	55.4 (53.3–57.6)	67.7 (65.6–69.9)	<.01
High school graduate or less (n = 1,463)	44.6 (42.4–46.7)	32.3 (30.1–34.4)	
**Poverty prevalence, %^c^**			
<20 (n = 2,924)	69.6 (67.7–71.6)	78.5 (76.3–80.5)	<.01
≥20 (n = 997)	30.4 (28.4–32.3)	21.5 (19.5–23.4)	
**% Non-Hispanic White**			
>50 (n = 3,347)	86.6 (85.1–88.1)	83.7 (82.0–85.4)	.01
≤50 (n = 574)	13.4 (11.9–14.9)	16.3 (14.6–18.0)	

a Values may not sum to total because of missing data.

b Determined by using χ^2^ test.

c Percentage of the population living below the federal poverty line.

Prevalence of approaches to encourage supermarkets or full-service grocery stores to open did not significantly change between 2014 and 2021, and approximately 30% of municipalities reported at least 1 support during both survey years (Table 2). The most commonly reported support in this domain was tax incentives, which were reported by 21.8% of municipalities in 2021 and 20.5% of municipalities in 2014. In 2021, 19.5% of municipalities with supermarket policies explicitly prioritized low-income or under-resourced areas as part of their policies, not significantly different than 15.8% in 2014 (data not shown).

**Table 2:** Prevalence of Healthy Food Retail and Breastfeeding Supports Among US Municipalities, National Survey of Community-Based Policy and Environmental Supports for Healthy Eating and Active Living (CBS-HEAL), 2014 and 2021^a^

Support type	Survey year, 2014	Survey year, 2021	*P* value
	% (95% CI)		
**Healthy food retail**			
**Does your local government currently use any of the following approaches to encourage supermarkets and other full-service grocery stores to open stores? (n = 3,921)**			
Tax incentives (eg, tax abatement, tax credit, or property tax exemption)	20.5 (18.7–22.2)	21.8 (19.9–23.7)	.32
Grant or loan programs	11.8 (10.4–13.2)	13.4 (11.9–15.0)	.13
Programs to link store openings to broader neighborhood revitalization projects	12.0 (10.6–13.5)	11.0 (9.6–12.5)	.35
At least 1 of the above supports for supermarkets	30.7 (28.7–32.7)	28.6 (26.5–30.6)	.16
**Does your local government provide any of the following to help convenience or corner stores sell healthier foods? (n = 3,921)**			
Grant or low-interest loan programs to purchase equipment for storage or sales of healthful foods (eg, refrigeration or a point of sale system)	3.4 (2.6–4.2)	4.2 (3.3–5.1)	.23
Technical assistance or training programs to increase the ability to sell healthier foods (eg, support for new point of sale systems, marketing assistance, produce handling training, product placement)	3.7 (2.9–4.5)	2.5 (1.8–3.2)	.04
Programs to link convenience or corner store improvements to broader neighborhood revitalization projects (improvements to lighting, signage, safety, walkability)	10.5 (9.2–11.9)	5.6 (4.5–6.6)	<.01
At least 1 of the above supports for convenience/corner stores	13.4 (11.9–14.9)	8.6 (7.3–9.9)	<.01
**Does your local government have any policies related to farmers markets, farm stands, or green/produce carts that . . . (n = 3,921)**			
Allow vendors to sell fresh produce on city-owned property	59.6 (57.5–61.8)	54.5 (52.2–56.8)	<.01
Streamline processes for obtaining health and food safety permits and licenses	18.7 (16.9–20.4)	22.4 (20.5–24.4)	<.01
Extend waivers of required business permits or retail licensing fees or taxes	13.6 (12.1–15.1)	15.2 (13.6–16.9)	.15
Provide funds or in-kind services for personnel, signage, or advertising	15.5 (13.9–17.1)	14.0 (12.4–15.6)	.21
At least 1 of the above supports for farmers markets	64.3 (62.2–66.4)	60.1 (57.9–62.4)	.01
**Does your local government provide funding for electronic benefits transfer (EBT) machines or provide technical assistance on how to obtain or use EBT machines at local farmers markets, farm stands, or green/produce carts?^b^** **(N = 3,063; n = 1,590 in 2014; n = 1,473 in 2021)**	7.5 (6.2–8.8)	11.2 (9.6–12.9)	<.01
**Breastfeeding**			
Is there a policy that allows all breastfeeding employees in the local government breaktime and space to pump breast milk? (% yes) (n = 3,954)	25.2 (23.3–27.1)	52.1 (49.8–54.4)	<.01^c^
Does your local government provide paid maternity leave for its employees? (% yes) (n = 3,954)	37.4 (35.9–39.5)	38.5 (36.3–40.8)	.47^c^
Does your local government provide ≥ 8 weeks of paid maternity leave for employees? (% yes) (n = 3,954)	15.5 (13.9–17.1)	18.9 (17.1–20.7)	.01^c^
Does your local government provide >12 weeks of paid maternity leave for employees? (% yes) (n = 3,954)	2.1 (1.5–2.8)	2.7 (2.0–3.4)	.30^c^

a Sample sizes for food retail support: n = 1,994 in 2014 and n = 1,927 in 2021. Sample sizes for breastfeeding support: n = 2,014 in 2014 and n = 1,940 in 2021.

b Among municipalities that currently have or formerly had farmers markets.

c *P* values for breastfeeding support determined using χ^2^ test.

Supports to help convenience or corner stores sell healthier foods were less commonly reported than supermarket supports, and the percentage of municipalities reporting at least 1 convenience store support decreased from 13.4% in 2014 to 8.6% in 2021 (Table 2). Programs to link convenience or corner store improvements to broader neighborhood revitalization projects were the most common convenience store support in 2021 (5.6%) but had decreased significantly from 2014 (10.5%). In 2021, 28.9% of municipalities with convenience or corner store policies explicitly prioritized low-income or under-resourced areas as part of their policies, a prevalence not significantly different from 21.4% in 2014 (data not shown).

Supports for farmers markets were the most commonly reported domain in both 2014 and 2021 although the prevalence of at least 1 farmers market support decreased significantly from 64.3% to 60.1% during that time (Table 2). Allowing vendors to sell fresh produce on city-owned property was the most commonly reported support in this domain but decreased from 59.6% in 2014 to 54.5% in 2021 (*P* < .01). Streamlining processes for obtaining health and food safety permits and licenses was the second most common farmers market support and increased (*P* < .01) from 18.7% to 22.4% between survey years. Among municipalities with farmers markets, the proportion that provided funding or technical assistance to farmers markets for electronic benefits transfer (EBT) increased from 7.5% in 2014 to 11.2% in 2021 (*P* < .01).

From 2014 to 2021, the percentage of municipalities with a policy allowing all government employees who were breastfeeding break time and space to pump breast milk more than doubled from 25.2% to 52.1% (*P* < .001) (Table 2). Although the prevalence of municipalities offering any paid maternity leave to employees did not significantly change from 2014 to 2021 (37.4% vs 38.5%), the percentage of those who offered 8 or more weeks of paid maternity leave increased from 15.5% to 18.9% (*P* = .01). However, the percentage of municipalities that provided ≥12 weeks did not significantly increase and remained between 2% and 3%.

Overall, the prevalence of having at least 1 supermarket, convenience store, and farmers market support policy remained stable or somewhat declined from 2014 to 2021 across municipality characteristics (Table 3). Significant decreases from 2014 to 2021 in the prevalence of having at least 1 supermarket policy were observed among medium-sized municipalities (33.6% to 29.2%, *P* = .03), Northeastern municipalities (23.8% to 16.2%, *P* = .03), municipalities with higher median educational attainment (31.3% to 27.5%, *P* = .047), and those with lower poverty prevalence (30.8% to 26.9%, *P* = .03). Regarding convenience store policies, significant declines over time were observed in small- (8.5% to 5.3%, *P* = .02) and medium-sized municipalities (14.3% to 9.0%, *P* < .01), urban municipalities (14.9% to 9.6%, *P* < .01), and in each Census region except the Northeast. Declines were also observed among those with both high and lower median educational attainment, high and lower poverty prevalence, and in both majority and minority non-Hispanic White municipalities. Farmers market supports decreased among medium sized municipalities (69.7% to 63.4%, *P* < .01), urban municipalities (66.5% to 61.4%, *P* = .01), municipalities with higher median educational attainment (65.3% to 60.7%, *P* = .02), and in majority non-Hispanic White municipalities (64.4% to 60.1%, *P* = .01).

**Table 3:** Prevalence of at Least 1 Policy to Support Healthy Food Access in Supermarkets, Convenience Stores, and Farmers Markets According to Municipality Characteristics; National Survey of Community-Based Policy and Environmental Supports for Healthy Eating and Active Living (CBS-HEAL) 2014 and 2021

Municipality characteristic	Supermarket policy^a^			Convenience store policy^b^			Farmers market policy^c^		
	% (95% CI)		χ^2^ *P* value^d^	% (95% CI)		χ^2^ *P* value^d^	% (95% CI)		χ^2^ *P* value^d^
	2014	2021		2014	2021		2014	2021	
**Population size**									
1,000–2,499 (n = 1,367)	24.9 (21.7–28.0)	24.2 (20.9–27.7)	.80	8.5 (6.5–10.6)	5.3 (3.6–7.1)	.02	53.4 (49.7–57.1)	50.4 (46.4–54.4)	.29
2,500–49,999 (n = 2,259)	33.6 (30.9–36.4)	29.2 (26.4–31.9)	.03	14.3 (12.3–16.4)	9.0 (7.2–10.7)	<.01	69.7 (67.0–72.4)	63.4 (60.4–66.3)	<.01
≥50,000 (n = 295)	35.2 (27.2–43.2)	43.7 (35.8–51.7)	.14	30.5 (22.7–38.2)	21.0 (14.5–27.5)	.07	73.7 (66.5–81.0)	79.6 (73.2–86.0)	.23
**Rural–urban status**									
Urban (n = 2,875)	31.9 (29.5–34.3)	28.7 (26.3–31.1)	.07	14.9 (13.0–16.7)	9.6 (8.0–11.1)	<.01	66.5 (64.1–69.0)	61.4 (58.8–64.0)	.01
Rural (n = 1,038)	27.0 (23.3–30.7)	28.0 (23.9–32.1)	.73	9.0 (6.5–11.4)	5.8 (3.7–7.9)	.06	57.6 (53.4–61.8)	56.3 (51.7–60.9)	.68
**Census region**									
Northeast (n = 523)	23.8 (18.3–29.4)	16.2 (11.8–20.7)	.03	15.2 (10.5–19.8)	10.5 (6.9–14.2)	.12	62.1 (55.8–68.3)	53.4 (47.3–59.5)	.05
Midwest (n = 1,383)	41.6 (38.0–45.2)	41.4 (37.6–45.2)	.94	14.1 (11.6–16.6)	9.9 (7.6–12.2)	.02	66.7 (63.3–70.2)	62.7 (59.0–66.5)	.12
South (n = 1,239)	27.8 (24.4–31.1)	25.5 (21.9–29.2)	.39	12.5 (10.0–15.0)	7.1 (4.9–9.2)	<.01	61.8 (58.2–65.4)	59.3 (55.2–63.4)	.38
West (n = 776)	18.4 (14.2–22.6)	16.8 (13.2–20.3)	.56	12.1 (8.6–15.6)	7.3 (4.9–9.8)	.03	66.7 (61.6–71.8)	62.4 (57.8–66.9)	.21
**Median educational attainment**									
≥Some college (n = 2,458)	31.3 (28.5–34.0)	27.5 (25.1–30.0)	.047	13.7 (11.6–15.7)	8.9 (7.3–10.4)	<.01	65.3 (62.5–68.1)	60.7 (58.0–63.4)	.02
≤High school graduate (n = 1,463)	29.9 (26.9–33.0)	30.8 (26.9–34.6)	.75	13.1 (10.8–15.3)	8.1 (5.8–10.3)	<.01	63.0 (59.8–66.2)	59.0 (54.9–63.1)	.13
**Poverty prevalence^e^**									
<20% (n = 2,924)	30.8 (28.4–33.2)	26.9 (24.7–29.2)	.03	12.2 (10.5–14.0)	8.3 (6.9–9.7)	<.01	62.3 (59.7–64.8)	59.4 (56.8–61.9)	.12
≥20% (n = 997)	30.4 (26.7–34.1)	34.5 (29.6–39.4)	.19	16.0 (13.1–19.0)	9.7 (6.7–12.6)	<.01	68.9 (65.2–72.6)	63.0 (58.1–68.0)	.06
**% Non-Hispanic White**									
>50 (n = 3,347)	30.3 (28.1–32.4)	27.7 (25.4–29.9)	.11	12.4 (10.8–14.0)	7.9 (6.6–9.2)	<.01	64.4 (62.1–66.7)	60.1 (57.6–62.6)	.01
≤50 (n = 574)	33.3 (27.6–39.0)	33.1 (27.6–38.5)	.95	16.7 (14.8–24.5)	12.2 (8.5–16.0)	.02	63.3 (57.5–69.1)	60.5 (54.9–66.2)	.50

a One or more of the following policies for supermarkets: tax incentives, grant or loan programs, or programs to link store openings to broader neighborhood revitalization projects.

b One or more of the following policies for convenience stores: grant or low-interest loan programs to purchase equipment for storage or sales of healthful foods, technical assistance or training programs to increase the ability to sell healthier foods, or programs to link convenience or corner store improvements to broader neighborhood revitalization project.

c One or more of the following policies for farmers markets: allowing vendors to sell fresh produce on city-owned property, streamlining processes for obtaining health and food safety permits and licenses, waivers of required business permits or retail licensing fees or taxes, or providing funds or in-kind services for personnel, signage, or advertising.

d Chi-square tests significance of difference between prevalence in 2014 vs 2021 for each level of each municipal characteristic.

e Percentage of the population living below the federal poverty line.

Regarding breastfeeding policy supports, providing any paid maternity leave to all government employees increased only among large municipalities (41.1% to 53.2%, *P* = .04) (Table 4). Providing 8 or more weeks paid maternity leave increased among urban municipalities (17.3% to 21.3%, *P* = .01), Northeastern municipalities (12.2% to 19.6%, *P* = .03), those with higher poverty prevalence (14.9% to 21.7%, *P* = .01), and those where most of the population was not non-Hispanic White (15.1% to 18.1%, *P* = .02). Providing 12 or more weeks paid maternity leave increased only in the West (3.0% to 7.8%, *P* = .01). Regarding policies to provide breaktime and space to pump breast milk, significant increases in prevalence between 2014 and 2021 were observed across all municipal characteristics. For example, prevalence more than tripled among small municipalities (13.2% to 42.3%, *P* < .01), while substantial increases were also observed among both medium (29.2% to 55.5%, *P* < .01) and large municipalities (52.6% to 71.7%, *P* < .01). Likewise, prevalence more than doubled in every Census region except the West, which still increased substantially from 45.1% to 67.3%.

**Table 4:** Prevalence of Policies to Support Breastfeeding Among Local Government Employees According to Municipality Characteristics, National Survey of Community-Based Policy and Environmental Supports for Healthy Eating and Active Living (CBS-HEAL), 2014 and 2021

Municipality characteristic	Provide paid maternity leave			Provide ≥8 weeks paid maternity leave			Provide ≥12 weeks paid maternity leave			Provide breaktime and space to pump breast milk		
	% (95% CI)		χ^2^ *P* value^a^	% (95% CI)		χ^2^ *P* value^a^	% (95% CI)		χ^2^ *P* value^a^	% (95% CI)		χ^2^ *P* value^a^
	2014	2021		2014	2021		2014	2021		2014	2021	
**Population size**												
1,000–2,499 (n = 1,383)	32.0 (28.5–35.4)	30.6 (26.9–34.3)	.60	9.0 (6.9–11.1)	11.8 (9.2–14.4)	.10	0.9 (0.2–1.5)	1.1 (0.3–2.0)	.60	13.2 (10.8–15.7)	42.3 (38.3–46.2)	<.01
2,500–49,999 (n = 2,273)	40.2 (37.4–43.1)	41.4 (38.4–44.3)	.59	18.1 (15.9–20.3)	20.9 (18.5–23.3)	.09	2.6 (1.7–3.5)	3.0 (2.0–4.0)	.58	29.2 (26.6–31.8)	55.5 (52.5–58.5)	<.01
≥50,000 (n = 298)	41.1 (33.0–49.3)	53.2 (45.2–61.1)	.04	26.9 (19.6–34.2)	35.9 (28.3–43.5)	.10	5.0 (1.3–8.6)	7.2 (3.4–11.0)	.41	52.6 (44.3–60.9)	71.7 (64.4–79.0)	<.01
Rural–urban status												
Urban (n = 2,897)	38.7 (36.2–41.2)	41.2 (38.6–43.8)	.18	17.3 (15.3–19.2)	21.3 (19.2–23.5)	.01	2.6 (1.7–3.4)	3.3 (2.4–4.2)	.23	28.9 (26.6–31.2)	55.4 (52.7–58.0)	<.01
Rural (n = 1049)	33.5 (29.5–37.5)	31.0 (26.8–35.3)	.40	10.4 (7.8–13.0)	11.7 (8.8–14.7)	.50	0.9 (0.1–1.8)	0.7 (0.0–1.6)	.74	14.4 (11.5–17.4)	42.3 (37.8–46.9)	<.01
Census region												
Northeast (n = 525)	29.4 (23.6–35.3)	36.3 (30.4–42.1)	.11	12.2 (7.9–16.4)	19.6 (14.7–24.4)	.03	2.2 (0.3–4.1)	3.5 (1.2–5.8)	.39	16.9 (12.0–21.7)	44.6 (38.6–50.7)	<.01
Midwest (n = 1,398)	39.1 (35.7–42.7)	35.7 (32.0–39.4)	.18	15.5 (12.9–18.1)	15.6 (12.8–18.4)	.95	1.6 (0.7–2.5)	1.1 (0.3–1.9)	.42	24.7 (21.6–27.8)	52.1 (48.3–56.0)	<.01
South (n = 1,256)	36.8 (33.2–40.4)	38.6 (34.5–42.6)	.52	14.2 (11.6–16.8)	18.3 (15.1–21.5)	.05	2.3 (1.2–3.4)	1.8 (0.7–2.8)	.50	21.1 (18.1–24.1)	48.9 (44.8–53.1)	<.01
West (n = 775)	42.6 (37.3–47.9)	47.5 (42.8–52.2)	.18	22.3 (17.8–26.7)	27.6 (23.3–31.8)	.10	3.0 (1.2–4.9)	7.8 (5.2–10.4)	.01	45.1 (39.7–50.4)	67.3 (63.0–71.7)	<.01
Median educational attainment												
≥Some college (n = 2,476)	39.2 (32.3–42.0)	39.3 (36.6–42.0)	.93	17.1 (14.9–19.3)	20.0 (17.8–22.2)	.07	2.3 (1.4–3.2)	2.7 (1.9–3.6)	.49	30.1 (27.5–32.8)	53.6 (53.6–59.1)	<.01
≤High school graduate (n = 1,478)	35.2 (32.1–38.4)	36.9 (32.9–40.9)	.52	13.5 (11.3–15.8)	15.6 (13.5–19.7)	.11	2.0 (1.1–2.9)	2.5 (1.2–3.7)	.50	19.1 (16.5–21.7)	43.3 (39.2–47.4)	<.01
Poverty prevalence^b^												
<20% (n = 2,950)	37.5 (35.0–40.1)	37.6 (35.1–40.1)	.98	15.8 (13.9–17.7)	18.1 (16.4–20.1)	.10	2.2 (1.4–2.9)	2.6 (1.8–3.4)	.43	26.3 (24.0–28.5)	53.2 (50.6–55.8)	<.01
≥20% (n = 1,004)	37.1 (33.3–40.9)	42.0 (37.0–47.0)	.13	14.9 (12.1–17.7)	21.7 (17.5–25.9)	.01	2.1 (1.0–3.2)	2.8 (1.2–4.4)	.46	22.8 (19.5–26.1)	48.2 (43.1–53.3)	<.01
% Non-Hispanic White												
>50% (n = 3,377)	36.6 (34.4–38.9)	36.6 (34.2–39.1)	.99	18.2 (13.6–22.8)	22.9 (18.1–27.6)	.17	2.1 (1.4–2.7)	2.1 (1.4–2.8)	.93	25.1 (23.0–27.1)	52.4 (49.9–54.9)	<.01
≤50% (n = 577)	42.4 (36.4–48.4)	48.2 (42.5–54.0)	.17	15.1 (13.4–16.8)	18.1 (16.2–20.0)	.02	2.7 (0.7–4.6)	5.4 (3.0–7.8)	.10	26.3 (21.0–31.6)	50.9 (45.1–56.6)	<.01

a Chi-square tests significance of difference between prevalence in 2014 vs 2021 for each level of each municipal characteristic.

b Percentage of the population living below the federal poverty line.

In sensitivity analyses (not shown) where “don’t know” responses were excluded from overall prevalence estimates and χ^2^ tests, results remained similar with a few exceptions. Specifically, tax incentives to encourage supermarket openings increased significantly from 18.7% in 2014 to 22.4% in 2021 (*P* = .02). Two previously significant declines in prevalence from 2014 to 2021 were no longer significant: technical assistance or training programs to increase the ability to sell healthier foods in convenience stores (2.8% vs 2.5%; *P* = .64) and the prevalence of 1 or more policies to support farmers markets (62.9% vs 59.6%; *P* = .07).

## Discussion

Our findings suggest that municipal-level policy supports for healthy food retail in supermarkets, convenience stores, and farmers markets did not increase in prevalence between 2014 and 2021, with prevalence of most policies remaining either unchanged or decreasing slightly. However, prevalence of policy supports for government employees who were breastfeeding increased substantially during the same time. In particular, the prevalence of policies to allow all breastfeeding employees time and space to pump breast milk doubled from approximately one-quarter of municipalities in 2014 to more than half in 2021 with significant increases observed across municipalities of all sizes and regions. Furthermore, the percentage of municipalities that offered 8 or more weeks of maternity leave increased significantly overall.

Our findings on the prevalence of healthy food retail policies suggest that the motivation to implement such policies may have waned or been surpassed by other policy priorities since 2014. For example, during 2020 and 2021, many local governments were likely occupied by responding to the COVID-19 pandemic, making food access policies through retail venues less of a priority (11). It is also possible that local governments may have been seeking different types of policies to improve access to healthy food. For example, it has been posited that focusing only on the distance to supermarkets and the need for more supermarkets may oversimplify the concept of access, which also encompasses transportation and the economic means to purchase healthy foods (12). In addition, evaluations of corner store initiatives have demonstrated mixed effectiveness in increasing fruit and vegetable availability, purchasing, or consumption (13,14). Reviews of the evidence evaluating efforts to increase supermarket access also suggest that such efforts may be of limited effectiveness (15). Nonetheless, evidence exists that encouraging supermarkets to open or remain open in food desert areas can provide access to healthy foods to local residents as well as employment and economic benefits to the local communities (16), and several studies have found modest effects of changes in supermarket or convenience store access on reducing children’s weight gain in low-income urban settings (17,18). Furthermore, evidence exists that access to farmers markets may be associated with increased purchasing or consumption of fruits and vegetables (19,20). Taken together, improving access to healthy foods in the retail food environment may require more complex and comprehensive efforts rather than focusing on one type of store, and some organizations have proposed a systems approach to improve healthy food access in communities (21). Future studies may seek to examine how to better address and improve healthy food access in communities using such a systems approach.

Our study suggests that the prevalence of some policies that support breastfeeding among municipal employees increased between 2014 and 2021. Breastfeeding has numerous short- and long-term health benefits for both children and mothers (22), and the Dietary Guidelines for Americans 2020–2025 recommend exclusive breastfeeding for 6 months followed by continued breastfeeding to complement solid foods until age 1 or longer (2). Workplaces have been recognized as important settings to support breastfeeding among working mothers (23,24). Worksite interventions to support breastfeeding, including providing break time and a space for nursing mothers to breastfeed or pump breast milk, have demonstrated improved breastfeeding duration and exclusive breastfeeding outcomes (24). The Breaktime for Nursing Mothers provision under the Fair Labor Standard Act (Section 7 of the FLSA) requires employers to provide reasonable breaktime and a private space, other than a bathroom, for hourly employees to express breast milk for 1 year after a child’s birth (25). While employees that fall within certain FLSA job categories (eg, executive, administrative, and professional employees) were not covered by this law, employers could have chosen to develop a policy that provides these benefits to all employees. Nonetheless, despite improvements observed in this study, only half of municipalities had such a policy in 2021, suggesting further room for improvement in supporting breastfeeding among local employees.

The prevalence of any paid maternity leave for all municipal employees only increased among large US municipalities but did not change significantly among US municipalities overall between 2014 and 2021, with approximately 37% to 39% of municipal governments offering it to their employees. This finding is consistent with the 40% prevalence observed among US employers in general (26) and suggests that more than 60% of municipalities do not offer any paid maternity leave. The Family and Medical Leave Act requires covered employers to provide unpaid maternity or medical leave but does not require employers to provide paid maternity leave (27). We did observe an increase from 2014 to 2021 in the prevalence of municipalities offering 8 or more weeks of paid maternity leave, but still only approximately 1 in 5 municipalities offer this. Paid maternity leave has been associated with improved breastfeeding outcomes with further improvements observed with increased duration of maternity leave (28). One study showed a modest increase in exclusive breastfeeding at 6 months with 6 weeks or more of paid family leave (29). Additional studies showed that a shorter duration of breastfeeding was associated with leave, either paid or unpaid, of less than 12 weeks (30). In our study, only 2% to 3% of municipalities offered 12 or more weeks of paid maternity leave. Although increased prevalence was observed among municipalities in the West, prevalence among every subgroup of municipalities was less than 10%. Thus, there remains a large opportunity for municipal governments to improve this breastfeeding support for their employees.

The CBS-HEAL study is the only nationally representative survey of US municipalities regarding policies and practices that support healthy eating and active living. Nonetheless there are some limitations to our study. First, because the survey relies on self-report of the respondent, we are unable to confirm whether a reported policy exists officially or has been implemented. Second, approximately half of eligible municipalities participated in the study and although we accounted for nonresponse in our sample weights, it is possible that some nonresponse bias still exists. Third, the frequency of “don’t know” responses ranged from 6% to 15% for food retail supports and was nearly 25% for the breastfeeding support policy for space and time to pump breast milk. Since we coded “don’t know” as “no,” it is likely that our prevalence estimates may underestimate the true prevalence of each policy because some municipalities that responded “don’t know” may have such a policy. However, our sensitivity analysis suggests that overall changes in healthy food retail and breastfeeding support policies observed in this study are unlikely to be the result of changes in “don’t know” responses between the 2 survey periods. Finally, because federal law already requires lactation accommodation for hourly employees, it is possible that local jurisdictions may accommodate all employees but have not written this into policy.

In conclusion, we found that the prevalence of healthy food retail supports for supermarkets, convenience stores, and farmers markets generally did not increase among US municipalities between 2014 and 2021, while policy supports for breastfeeding among municipal employees increased substantially during this time. Opportunities remain for municipalities to support healthy eating and breastfeeding among residents and employees.
